# Knowledge and experience of a cohort of HIV-positive and HIV-negative Ghanaian women after undergoing human papillomavirus and cervical cancer screening

**DOI:** 10.1186/s12905-019-0818-y

**Published:** 2019-10-23

**Authors:** Arabella Stuart, Dorcas Obiri-Yeboah, Yaw Adu-Sarkodie, Anna Hayfron-Benjamin, Angela D. Akorsu, Philippe Mayaud

**Affiliations:** 1 University Hospital, Lewisham, London, UK; 2 Department of Clinical Research, Faculty of Infectious and Tropical Diseases, London School of Hygiene and Tropical Medicine, London, UK; 3 Department of Microbiology and Immunology, School of Medical Sciences, CoHAS, University of Cape Coast, Private Mail Bag, Cape Coast, Ghana; 4 Department of Clinical Microbiology, School of Medical Sciences, Kwame Nkrumah University of Science and Technology, Kumasi, Ghana; 5 Department of Maternal and Child Health, School of Nursing and Midwifery, University of Cape Coast, Cape Coast, Ghana; 6 Institute of Development Studies, University of Cape Coast, Cape Coast, Ghana

**Keywords:** HPV, Cervical cancer, Knowledge, HIV, Africa, Qualitative research, Quantitative research

## Abstract

**Background:**

Cervical cancer is the most common cancer in women in Ghana, but knowledge and experience of women who have had cervical screening is under-evaluated. This study examined knowledge and understanding of HPV and cervical cancer and evaluated experiences of screening in a cohort of women of mixed HIV status.

**Methods:**

This was a mixed methods study using questionnaires and focus group discussions, with a knowledge score constructed from the questionnaire. HIV-positive and HIV-negative women were recruited from a larger cervical screening study in Ghana and were interviewed 6 months after receiving screening. Quantitative data was analyzed and triangulated with qualitative data following thematic analysis using the framework approach.

**Results:**

A total of 131 women were included (HIV-positive, *n* = 60). Over 80% of participants had a knowledge score deemed adequate. There was no difference between HIV-status groups in overall knowledge scores (*p* = 0.1), but variation was seen in individual knowledge items. HIV-positive women were more likely to correctly identify HPV as being sexually-transmitted (*p* = 0.05), and HIV negative women to correctly identify the stages in developing cervical cancer (p = < 0.0001).

HIV-positive women mostly described acquisition of HPV in stigmatising terms. The early asymptomatic phase of cervical cancer made it difficult for women to define “what” cancer was versus “what” HPV infection was. All women expressed that they found it difficult waiting for their screening results but that receiving information and counselling from health workers alleviated anxiety.

**Conclusions:**

Knowledge of women who had participated in a cervical screening study was good, but specific misconceptions existed. HIV-positive women had similar levels of knowledge to HIV-negative, but different misconceptions. Women expressed generally positive views about screening, but did experience distress. A standardized education tool explaining cervical screening and relevance specifically of HPV-DNA results in Ghana should be developed, taking into consideration the different needs of HIV-positive women.

## Background

Cervical cancer is responsible for the highest number of cancer-related deaths among women in Ghana, with 3052 new cases and 1556 deaths annually [1]. HPV vaccines are licenced for use, but have not been introduced beyond a pilot program in selected regions commenced in 2013 [2]. Ghana has had a national policy on cervical cancer prevention since 2005, which recommends screening with visual inspection with acetic acid (VIA) and treatment of lesions with cryotherapy for women between the ages of 25–45 years, and papanicolau (PAP) smears for women over the age of 45 [3]. The National Screening Program so far has limited coverage of estimated 2.7% of the eligible population. Both Papanicolaou smears and VIA are available in public and private health clinics at a cost to the patient since the national health insurance does not cover it.

HIV infection is an established co-factor in the development of cervical cancer as it increases susceptibility to persistent HPV infection [4, 5]. Women living with HIV have an incidence of cervical cancer seven times that of women not infected with HIV, risk developing disease up to 10 years earlier, and require more frequent screening [4, 6].

Non-attendance at cervical screening remains a significant problem in high-income countries with established programmes, with an estimated 50–60% of cervical cancers occurring in women who have never attended screening [7, 8]. Poor knowledge about the disease and the benefits of screening have been shown to be associated with non-attendance [9, 10]. Barriers to women attending screening include embarrassment related to sample collection, fear of pain, and fear of cancer diagnosis [11, 12].

Understanding the screening process and the benefits of early treatment are fundamental to women engaging in screening and follow-up care [10, 13]. This is especially important in low and middle-income countries (LMIC) where costs and accessibility may represent significant barriers to screening, as women are not incentivised to seek testing for what is often an asymptomatic condition [14].

Surveys of women across Sub-Saharan Africa (SSA) show great variation in women’s awareness and knowledge of cervical cancer [9, 15, 16]. A survey in Ethiopia found that 78.7% of women surveyed had heard of cervical cancer, but only 31% were deemed “knowledgeable” [17]. In a study in the Democratic Republic of Congo 81.9% of women surveyed had heard of cervical cancer, but only 43% were felt to have sufficient knowledge [18]. Surveys of market women in Nigeria, and of women in health-facilities in Kenya found only 6.9 and 29% of respondents, respectively, having heard of cervical cancer [15, 19].

In Ghana there is also evidence of this variation, with a survey of university students finding 93% of respondents aware of cervical cancer in comparison to a survey of women from the general population which found that 68.4% had never heard of cervical cancer, 93.6% were not aware of the risk factors, and 97.7% had not heard of cervical screening [9, 20].

Few studies in SSA have evaluated the knowledge of women undergoing cervical cancer screening [21, 22], but none has investigated the experience of women after undergoing screening, and none have included women living with HIV. The advent of HPV-DNA testing in high-income countries has changed the experience of cervical screening, with issues such as increased health-anxiety, stigma of HPV infection, and confusion around health information arising [23–25]. HPV-DNA testing is now being used in conjunction with cytology in many high-income countries, and has been adopted as the primary screening method in some [26, 27]. A number of trials have evaluated the use of HPV-DNA testing in low-resource environments, and have shown high sensitivity for the detection of cervical intraepithelial neoplasia (CIN) grades 2 and above, in both human immunodeficiency virus (HIV) seropositive and HIV-negative women [28, 29]. Experience of HPV-DNA testing has not yet been evaluated in Ghana or other SSA countries as testing is not widely available. This area needs exploration as low-cost rapid HPV DNA tests suitable for use in the low resource setting have been developed and are expected to become more frequently used for cervical screening [29].

This study examined the knowledge and understanding of HPV, cervical cancer, and cervical cancer screening, and evaluated the experience of cervical screening in a cohort of HIV-positive and HIV-negative women who had received screening with a combination of cervical cytology and HPV-DNA testing as part of another research study [30–32]. The aim is to inform the development of effective screening messages, improve information provided to women around screening and evaluate whether women living with HIV have different information needs.

## Methods

### Study design and population

We conducted a mixed-methods study using convergent parallel design with quantitative and qualitative strands of the study implemented concurrently, kept independent during analysis and combined in interpretation [33]. We used a combination of interviewer-administered questionnaires and focus group discussions (FGDs).

Participants were sampled purposively from a parent-cohort study of women at the Cape Coast Teaching Hospital (CCTH) in Cape Coast, Ghana, (parent study *N* = 343, HIV-positive *n* = 173). The parent study was a comparative-cohort study of HIV-positive and HIV-negative women that investigated the epidemiology of HPV and cervical squamous intraepithelial lesions (SIL); evaluated a rapid HPV DNA screening test (*care*HPV, Qiagen, Gaithersburg, MD), and determined the performance and acceptability of self-sampling for HPV testing [30–32]. At parent-study enrolment, women underwent gynaecological examination, HPV-DNA testing and cervical cytology, and socio-demographic and medical history data were collected. During this first visit, they were also given basic facts about HPV and cervical cancer. At 6-months participants were followed up in clinic with review of symptoms, gynaecological examination, and repeat cervical cytology. Women found to have abnormal cytology after the first screening were referred for gynaecological evaluation and treatment. At the 6-month parent-study follow up visit participants were informed about the sub-study and consenting individuals consecutively recruited.

There was no sample size calculation as the sub-study was purely descriptive with no tested hypothesis. We aimed to collect a minimum of 100 questionnaires (50% HIV-positive). The inclusion criterion was participation in the parent-study (age > 18 years and accepting HIV testing); there were no exclusion criteria. Respondents were asked on the questionnaire if they would consider participating in a FGD; those who consented were called at random until 16 (half of each HIV serostatus) had been recruited.

### Quantitative methods

A 32-item questionnaire was developed specifically for this study through literature review and collaboration with the parent-study investigators. Information on knowledge (HPV, cervical cancer and screening) and screening experiences of the women was gathered. The questionnaire was piloted with administering study-personnel (four nurses and one doctor) prior to initiation. The questionnaire was developed in English, but administered face-to-face in the preferred language of the respondent. Clinic staff were fluent in local languages and English and there was no formal assessment of their language skills. Specific translations of each question into local languages were chosen during piloting. Demographic data, HPV and HIV testing results were extracted from the parent-study.

Descriptive analysis using frequencies and percentages with bivariate analysis by HIV-status and other participant characteristics was performed on categorical data. Likert-type item responses were grouped into “overall agree” “neutral” and “overall disagree” from a five point response scale of strongly disagree/disagree/neutral/agree/strongly agree. Chi-square tests or Fisher’s exact test were used to obtain *p-*values. A knowledge score was created from the knowledge items. Correct responses were scored as “1”, and incorrect or “unsure” responses as “0”. There were nine knowledge items in the questionnaire which participants had been exposed to during parent-study counselling and were defined as “expected” knowledge items. The parent study counselling was delivered verbally through the clinic nurses and doctor and was supplemented with an information leaflet as part of the consent. The remaining ten knowledge items were not covered by parent-study counselling and were thus not defined as “expected” areas of knowledge. Therefore, the cut-off for “adequate knowledge” was a score of ≥9 (maximum score 19), which included correct responses to any question, not just those that were defined as “expected”. Bivariate analysis of mean knowledge score by demographic variables was performed using t-tests and ANOVA. Univariable risk factor analyses for knowledge adequacy was performed and reported as odds ratios (ORs) with 95% confidence interval (CI). Free-text responses were presented descriptively. Data were analyzed using Stata13.1 (StataCorp, Texas, USA).

### Qualitative methods

Focus-group discussions were held after the questionnaire collection phase had ended, and were run in the School of Medical Sciences teaching building at CCTH. There was no financial incentive for participation, but light refreshments were served and costs of travel reimbursed. Participants were stratified into two FGDs by HIV-status (eight participants in each). The aim of this was to 1) reduce the risk of stigmatisation through inadvertent disclosure of HIV-status during discussions, and 2) examine differences/similarities in results. Participants were not informed that their group was of particular HIV-status, and were not aware of other members’ HPV or cytology results.

A semi-structured topic guide using pre-scripted open-ended questions was used, focusing on knowledge and understanding of HPV, cervical cancer and screening; and on participants’ experience of the screening process. Both FGDs were conducted in the local language (Fante) and each lasted approximately 1h. Two female facilitators fluent in local languages and with experience of conducting FGDs, were recruited from the Institute of Development Studies at the University of Cape Coast. FGDs were recorded onto digital audio recorders, and field notes recording interactions were taken. Transcripts were translated into English and transcribed verbatim, and checked for accuracy. Thematic analysis was done using the Framework Method [34]. After familiarization with the transcripts, initial codes were generated manually using an inductive approach. Transcripts were then imported into Nvivo10 (QSR International) and fully coded, with generation of additional codes as they emerged. A framework matrix was created, with themes and sub-themes generated from the codes making up the columns of the matrix, and cases (individual participants), the rows. Associations, explanations and relationships were explored through the framework.

## Results

Unless otherwise stated, numerical results in the text are presented ordered as HIV-negative, HIV versus positive. Findings from both quantitative and qualitative aspects are presented together. A total of 135 women (HIV-positive, *n* = 60) completed the questionnaire, and 16 participated in the FGDs. Participant characteristics by HIV-status are presented in Table 1*.* Variables with significant differences by HIV-status were distribution of ages between categories (*p* = 0.04), occupation (*p* < 0.0001), level of education (*p* < 0.001), relationship status (p < 0.001), cytology results (*p* = 0.02), and HPV result (*p* = 0.001).

**Table 1:** Characteristics of 131 questionnaire participants attending the Cape Coast Teaching Hospital, Ghana, by HIV-status

	HIV-negative (*n* = 76) (%*)	HIV-positive (*n* = 55)(%*)	*p* value
Mean age (SD), years	43.9 (11.4)	42.9 (8.4)	0.59^**^
Age, by category, years			
20–29	6 (7.9)	2 (3.6)	0.04^†^
30–39	26 (34.2)	16 (29.1)	
40–49	17 (22.4)	26 (47.3)	
50–59	22 (29)	8 (14.6)	
60–70	5 (6.6)	3 (5.5)	
Occupation			
Unemployed	4 (5.3)	3 (5.5)	0.000^†^
Unskilled work	38 (50.0)	48 (87.3)	
Skilled work	34 (44.7)	4 (7.3)	
Religion			
Christian	69 (90.8)	47 (88.5)	0.39^†^
Muslim	7 (9.2)	7 (12.7)	
Traditional	0 (0)	1 (1.8)	
Relationship status			
Current partner	58 (76.3)	26 (47.3)	< 0.001^‡^
No current partner	18 (23.7)	29 (52.7)	
Level of education			
< 6 years formal education	11 (14.5)	29 (52.7)	< 0.001^‡^
> 6 years formal education	65 (85.5)	26 (47.3)	
Mean number of children	2.3 (1.9)	2.7 (1.8)	0.22^**^
HPV result			
HPV positive	32 (42.1)	39 (70.9)	0.001^‡^
HPV negative	44 (57.9)	16 (29.1)	
Cytology result 0.005			
Positive (> = ASCUS)	1 (1.3)	6 (10.9)	0.02^‡^
Negative	75 (98.7)	49 (89.1)	

*ASCUS* atypical squamous cells of undetermined significance, *SD* standard deviation

^*^ percentages may not sum to 100 due to rounding

^**^ calculated using two sample t-test

^†^ calculated using Fisher’s exact test

^‡^ calculated using Chi-squared test

### HPV and cervical cancer related knowledge

Results of the knowledge domains are presented in Table 2. Questions with the highest proportion of correct responses for HIV negative versus HIV positive women include HPV being sexually transmitted (86.7100%), HPV being the cause of cervical cancer (91.9% vrs. 98.2%), condoms being partially protective (82.7% vrs. 94.6%), and cervical cancer being preventable (90.8% vrs. 94.6%). Questions with the highest proportion of incorrect responses also tended to be those with the highest proportions of “unsure” responses. Questions showing difference by HIV-status included whether HPV is sexually transmitted (*p* = 0.01) and whether cervical cancer is rare in Ghana (*p* = 0.005), with HIV-positive women giving more frequent correct answers. HIV-negative women were more often correct in identifying that cervical cancer has stages (p = < 0.001).

**Table 2:** Responses to questionnaire knowledge-items among women (*n* = 131)

	HIV-negative (%^*^) *N* = 76	HIV-positive (%^*^) *N* = 55	*p* value
HPV is sexually transmitted^♦^			
**True**	**86.7%**	**100.0%**	**0.01** ^†^
False	6.7%	0.0%	
Unsure	6.7%	0.0%	
HPV causes cervical cancer^♦^			
**True**	**91.9%**	**98.2%**	**0.2** ^†^
False	0.0%	0.0%	
Unsure	8.1%	1.8%	
HPV infection is rare: not many people have it^♦^			
True	20.0%	25.5%	0.06^‡^
**False**	**41.3%**	**21.8%**	
Unsure	38.6%	52.7%	
Cervical cancer is rare in Ghana^♦^			
True	33.3%	21.8%	0.005^‡^
**False**	**40.0%**	**23.6%**	
Unsure	26.7%	54.6%	
If there are women in your family (who are blood relatives) who have had cervical cancer, this means it is more likely to happen to you. ^♦^			
**True**	**66.7%**	**30.9%**	**< 0.001** ^†^
False	6.7%	3.6%	
Unsure	26.7%	65.5%	
Which of these do you think can be signs of cervical cancer?^~^			
**Bleeding after sex**	**15.9%**	**13.2%**	**–**
**Smelly discharge from the vagina**	**15.6%**	**13%**	
**Bleeding in between menstrual periods**^♦^	**12.7%**	**10.0%**	
Itching of the vagina	6.6%	7.1%	
**No symptoms**^♦^	**3.7%**	**2.2%**	
Cervical cancer can be prevented^♦^			
**True**	**90.8%**	**94.6%**	
False	1.3%	0%	0.8^†^
Unsure	7.9%	5.5%	
Only women who are having vaginal complaints should have cervical screening^♦^			
True	4.0%	3.6%	1.000^†^
**False**	**94.7%**	**94.6%**	
Unsure	1.3%	1.8%	
Men cannot be infected with HPV			
True	20.0%	25.5%	0.3^†^
**False**	**66.6%**	**69.1%**	
Unsure	13.3%	5.5%	
Condoms offer some protection from getting infected with HPV			
**True**	**82.7%**	**94.6%**	**0.1** ^†^
False	6.7%	3.6%	
Unsure	10.7%	1.8%	
Using herbs in the vagina makes you more likely to get cervical cancer			
True	36.8%	21.8%	0.03^†^ 0.03^†^
**False**	**7.9%**	**1.8%**	
Unsure	55.3%	76.4%	
Having an abortion or miscarriage makes you more likely to get cervical cancer			
True	22.4%	21.8%	0.6^†^
**False**	**13.2%**	**7.3%**	
Unsure	64.5%	70.9%	
Unless you are on a study like this one, you cannot get cervical cancer screening in Ghana			
True	13.3%	9.1%	0.8^†^
**False**	**72%**	**76.4%**	
Unsure	14.7%	14.6%	
There are no stages to cervical cancer; either you have it or you don’t			
True	29.7%	67.3%	< 0.001^†^
**False**	**51.4%**	**25.5%**	
Unsure	18.9%	7.3%	
Cervical cancer is always fatal, even if caught at the early stages			
True	19.2%	24.1%	0.7^**‡**^
**False**	**65.8%**	**59.3%**	
Unsure	15.1%	16.7%	

^*^percentages may not sum to 100 due to rounding

^†^Fisher’s exact test

^‡^Chi-squared test

^~^ multiple response item, presented as proportions of responses

^--^ *p* values not calculated; multiple response items

^♦^ denotes “expected knowledge item” – discussed in parent study information and counselling

Question responses in **bold** denote correct response

**Fig. 1:**
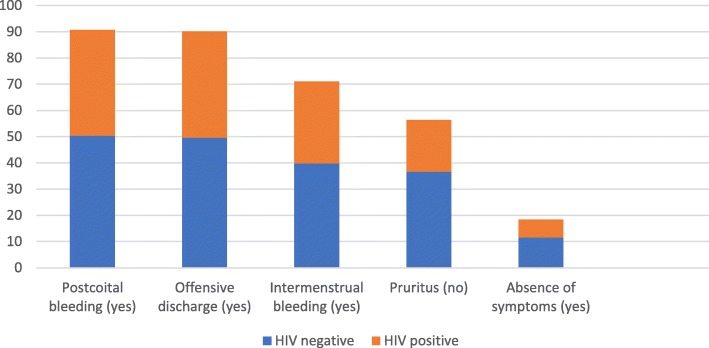
Proportion of women answering correctly which symptoms can be associated with cervical cancer, with breakdown by HIV-status among 131 women attending the Cape Coast Teaching Hospital, Ghana

The symptoms that survey participants associated with cervical cancer are detailed in Fig. 1. Postcoital bleeding, offensive vaginal discharge and intermenstrual bleeding were commonly correctly identified as potential symptoms (90.8, 90.1, and 70.1%). Pruritus was also a commonly misidentified as a potential symptom with 56.6% of participants selecting it. Identifying that one could still have cervical cancer but no symptoms was less common at 18.3%. More HIV-negative women (49.6% vrs. 40.5%, *p* = 0.04) correctly identified offensive vaginal discharge as a symptom, but there were no other statistically significant differences between HIV-status groups.

The mean knowledge score for all survey participants was 11.6 (SD + 2.7; range: 3–18), with no evidence of association with HIV-status (*p* = 0.1). An “adequate” knowledge score (correct responses for ≥9 of 19 items) was reached by 87.8% of participants, with no difference by HIV-status (*p* = 0.11). On bivariate analysis having a current partner was associated with having an adequate knowledge score (OR 2.53, 95%CI 1.03–6.24, *p* = 0.04).

Four themes relating to women’s knowledge of HPV, cervical cancer and screening were constructed from the FGDs: “acquisition”, “nature of disease”, “protection” and “testing”.

That HPV is sexually transmitted was commonly expressed, and the majority of women were clear on this with few incidences of misconceptions. The manner in which participants described the acquisition of HPV was categorised into “stigmatised” and “non-stigmatised”, with “stigmatised” expressions putting a critical or “moral” judgment on sexual transmission. These were more common amongst HIV-positive women, and were mostly related to the expression that sexual promiscuity was necessary for acquisition of disease:

*“The way through which we come to get it [HPV virus] is if we and men about three or four have sex.” (40-49yrs, HIV-positive, HPV-positive, cytology negative)*

“Non-stigmatised” acquisition was expressed in the form of simple statements that HPV is sexually transmitted. Only one participant mentioned HIV-status affecting vulnerability to infection:

*“I think when the doctor was teaching us she made me understand that it can attack everyone, but for those of us who specifically have HIV, it is easy that we can get it [HPV virus]” (30-39yrs, HIV-positive, HPV- and cytology-negative)*

The most common misconception expressed about acquisition was the effect of abortion (only expressed by HIV-negative participants):

*“I was told that women who cause abortions, those who have had cases of STDs, and those who have a lot of sexual partners are all at risk of getting infected.” (60-69yrs, HIV-negative, HPV-positive, cytology-negative)*

The early asymptomatic phase of cervical cancer made it more difficult for women to define “what” cancer was; for them cancer was something you could see or feel, and identify by characteristic symptoms. This also caused difficulty in identifying “what” HPV was, and in reconciling its lack of symptoms with its harmful nature:

*“At first, I thought cancer only affected the breast but we went for a workshop and heard that it can also affect the mouth of the womb, but that the cancer that affects the mouth of the womb has no symptoms because for the breast cancer you can see that your breast has lump in it and look some style (does not look normal) but they said for the cervical cancer it will be there without showing so we should start doing some test” (40-49yrs, HIV-positive, HPV- and cytology-negative)*

Only two (HIV-negative) women correctly expressed that HPV is an infection that can lead to cancer if it persists. When asked to describe the symptoms of cervical cancer and HPV they were again described interchangeably. Several women believed that HPV infection would cause vaginal discharge was expressed. One woman described the symptoms of advanced cervical cancer as:

*“vaginal discharge with very bad smell, abdominal pains, a lot of complications, loss of appetite, sores in the mouth, etc. The cancer affects every part of the body.” (50-59yrs, HIV-negative, HPV-positive, cytology-negative).*

A strong sub-theme of “nature of the disease” was the effect of HPV on men. Women queried whether it would also cause disease in men, or whether there would be any visible signs on men that they were infected with HPV:

*“Does it mean that this HPV it can be on [infect] males and it can be on females? But for the men when they have it doesn’t it show on their bodies or it does it not give them any problems?” (40-49yrs, HIV-positive, HPV- and cytology-negative*)

Some women expressed the belief that HPV would cause visible symptoms in an infected man:

*“Me too I think that if you are there with someone and he has it, you will see that his skin is changing” (40-49yrs, HIV-positive, HPV positive and cytology negative).*

There were also feelings of frustration from participants that men could engage in sexually “careless” behaviour without fear of consequences as:

*“the disease will affect you the woman” (60-69yrs, HIV-negative, HPV-positive, cytology-negative)*

Condoms were mentioned frequently and emphatically by participants as being important for protecting themselves and this was phrased in absolute terms*:*

*“I learnt that when you have sex and you protect yourself you will not be infected, usually through condom use.” (20-29yrs, HIV-negative, HPV- and cytology-positive).*

Modifying sexual behaviour by limiting one’s number of sexual partners, or exhibiting sexual ‘restraint’ was also expressed as an important factor in prevention, and was commonly referred to as “being careful” or “taking care”:

*“Eeeem … some people do not like condom so they will have to, reduce the men they do sex with and all that” (40-49yrs, HIV-positive, HPV- and cytology-negative)*

*“All we have to do is to take care of ourselves from this disease. Like pulling ourselves away from some things that when we do, it will not go and bring us this problem.” (30-39yrs, HIV-positive, HPV- and cytology-negative)*

Participants expressed feelings of powerlessness in protecting themselves from disease when discussing their partners. “Fidelity” and “sexual negotiation” were predominant sub-themes. Male partners’ infidelity was expected:

*“Because it is got from men, its prevention will be difficult. A woman cannot advise her husband to not have sex with other women.” (50-59yrs, HIV-negative, HPV- and cytology-positive).*

Women also believed that because in general men did know about HPV or cervical cancer they would not be taking any steps to reduce the risk of transmitting HPV to their female partners, and that education of men would be an important element in protecting women:

“*If education could be given on radio and TV for me, I believe it would help us.” (50-59yrs, HIV-negative, HPV-positive, cytology-negative)*

### Experience of screening

Responses to the Likert-type questionnaire items are presented in Table 3. Responses did not vary by HIV-status. Among HIV negative versus HIV positive women, a third of women (30.7% vrs. 35.0%) agreed that screening was embarrassing but most women did not find screening painful (85.3% vrs. 85.0%).

**Table 3:** Questionnaire responses on experience of screening, Likert-type items by HIV-status (*n* = 131)

	HIV-negative (%^*^) *n* = 76	HIV-positive (%^*^) *n* = 55	*p* value
“The screening was embarrassing”			
Disagree overall	50.7%	54.2%	0.4^‡^
Neutral	18.3%	10.2%	
Agree overall	31.0%	35.6%	
“The screening was painful”			
Disagree overall	84.5%	85.0%	1.0^†^
Neutral	5.6%	6.7%	
Agree overall	9.9%	8.3%	
“I was worried about the results of my screening”			
Disagree overall	31.4%	30.0%	0.8^‡^
Neutral	30.0%	26.7%	
Agree overall	38.6%	43.3%	
“I was given enough information about HPV, cervical cancer, and the screening test before the screening”			
Disagree overall	2.8%	1.7%	0.9^†^
Neutral	12.7%	10.0%	
Agree overall	84.5%	88.3%	

^*^percentages may not sum to 100 due to rounding

^†^Fisher’s exact test

^‡^Chi-squared test

The themes of “protection” and “testing” also emerged from the FGD aspects focussed on experience of screening, but the theme “fear and anxiety” was additionally constructed. Women were unanimous in their expression that learning about HPV and cervical cancer was frightening; and for many this related to simultaneously learning of the disease, and that they were at risk, for the first time:

*“Please, as for me it got me really scared, because me, myself, I had not heard some. Before I knew it is only breast that cancer affects, so me it got me very scared” (40-49yrs, HIV-positive HPV-positive, cytology-negative)*

Other elements that generated anxiety were: the “not knowing” that one could be infected with HPV due to its asymptomatic nature; what this meant for one’s relationship to one’s partner (and the question of infidelity); and media messages about cervical cancer:

*“What really pains me a little about it, is that when you get it you won’t get any symptoms that this is what is happening to you” (40-49yrs, HIV-positive, HPV- and cytology-negative)*

***“*** *The way the doctor said it on TV made it sound scary.” (40-49yrs, HIV-negative, HPV-positive, cytology-negative)*

When asked how they felt whilst waiting for their results, women commonly expressed that they found it difficult but receiving information and counselling from health workers alleviated fears and anxiety as well as the fact that testing would help discover any problems at an early stage:

*“I was scared, waiting for the result was very scary. I could not even sleep.” (60-69yrs, HIV-negative, HPV-positive, cytology-negative)*

*“It was scary at first but later I became relaxed. I also was glad about the fact that I took the test because then if there had been something it would be found and stopped.” (40-49yrs, HIV-negative, HPV-positive, cytology-negative).*

When asked if they had sought information from other sources between initial screening and follow up, under half said “yes” (41.4,40.0%). The source of information with the greatest proportion of responses was “other healthcare professional” at 45% of responses (Table 4.)

**Table 4:** Questionnaire responses: information seeking and impact of screening, by HIV-status

	HIV-negative (%^*^) *N* = 76	HIV-positive (%^*^) *N* = 55	*p* values
Did you seek information about HPV/cervical cancer/cervical screening from anywhere else between having the initial testing and coming back for follow up?			
Yes	41.4%	40.0%	0.9^‡^
If yes, where did you look for information^~^			
Friends/family	8.3%	12.5%	–
Doctors	22.2%	16.6%	
Other healthcare professional	44.4%	45.8%	
Internet	25.0%	25.0%	
Would you have cervical cancer screening again if it was free?			
Yes	100.0%	91.4%	0.02^†^
Would you have cervical cancer screening again if you had to pay for it?			
Yes	90.3%	80.7%	0.1^‡^
“I have told other women they should have cervical cancer screening”			
Yes	70.6%	70.2%	1.0^‡^

^*^percentages may not sum to 100 due to rounding

^~^multiple response item, presented as proportions of responses

^†^Fisher’s exact test

^‡^Chi-squared test

-- p values not calculated; multiple response items

Almost all women said they would have repeat cervical screening if it was free, with a statistically-significant difference between groups (100, 91.4%; *p* = 0.02). Of those who said they would have repeat cervical screening if it was free, 89.3% said they would also have it if there was a charge.

In FGDs women also talked about the cost of testing. Some women were aware of cervical screening prior to joining the parent-study but said they had not availed of it due to cost. It was also mentioned that the government should increase the availability of screening by reducing the cost:

*“The government should also try and reduce the cost for us” (50-59yrs, HIV-negative, HPV-positive, cytology-negative)*.

In FGDs, the view that screening was protective against developing disease through both informing and educating women and detecting disease early was frequently expressed:

*“If a test is done it would help prevent any further damage the disease would have caused to the womb if the result is positive. If it is negative, then you will be educated on how to stay safe or protect yourself.” (50-59yrs, HIV-negative, HPV-positive, cytology-negative)*

The sub-theme of “testing imperative” related to multiple expressions that testing was something that must be done if the opportunity presents itself; and that other women should seek testing. However, this was only voiced by women who had had a positive HPV result:

*“Whether morning or afternoon, wherever they call you for the test you will have to do it.” (40-49yrs, HIV-positive, HPV-positive, cytology-negative)*

*“This should be of greater concern to all women so that from time to time we can run the tests.” (40-49yrs, HIV-negative, HPV-positive, cytology-negative)*

## Discussion

In this study of mixed HIV-status women who had undergone cervical screening in Cape Coast, Ghana, we found good levels of knowledge of HPV, cervical cancer, and cervical screening, with 85–100% of participants able to correctly identify major factual points (HPV is sexually transmitted, HPV causes cervical cancer, cervical cancer can be prevented). Despite lower overall education status amongst HIV-positive women, there was no significant difference in mean knowledge score between groups. Specific misconceptions and attitudes to these subjects were highlighted through the FGDs, with stigmatising language used around the acquisition of HPV. Women had mixed experiences of the screening process, with around a third (HIV-negative: 30.7%, HIV-positive: 35.0%) finding it embarrassing, and a larger proportion (38.6% vrs. 43.3%) experiencing anxiety around their results. This anxiety element was echoed in the FGDs, but the positive aspects of screening and education provided by healthcare professionals were also highlighted.

Other studies in Ghana and SSA where women have not had specific health education has found knowledge of HPV, cervical cancer, and screening women to be poor [9, 16, 35]. This implies that for our cohort knowledge was gained through the parent-study counselling, but may have additionally been due to women being motivated to seek information: indeed, 40% of participants from both HIV-status groups sought information from other sources between initial screening and follow-up. The increased exposure of HIV-positive women to health education through regular HIV care may account for differences in knowledge responses, and also explain why lower levels of formal education in this group did not have an effect on knowledge scores.

Specific misconceptions were apparent in the FGDs, particularly in relation to the difference between HPV infection and cervical cancer. Many women expressed the belief that they were the same thing, and described them interchangeably. Participants rarely expressed knowledge of the latency period between HPV infection and development of cancer, contrasting with a Zambian study where this was frequently mentioned [36]. Overall, misconceptions appeared to be fewer than those found in other studies in SSA where vaginal hygiene practices, contraceptive and tampon use, witchcraft, benign vaginal infections, and “too much sex” were attributed causes of cervical cancer [15, 37, 38]. Whether misconceptions and knowledge gaps were due to the way in which study counselling was delivered, the interpretation of knowledge received, or pre-existing ideas and cultural concepts of disease is difficult to assess, but these findings have implications for the design of future education messages. Qualitative work in high-income populations has shown that screening can generate confusion for women with information from healthcare professionals, information leaflets and the internet often not meeting their needs [24, 39–41].

Stigma attached to the sexual transmission of HPV is a major theme in qualitative studies in many countries, and women usually frame this in the context of self-blame and shame for having “given themselves cancer” [23, 41, 42]. Women in our study expressed “stigmatised” statements that HPV infection was due to sexual promiscuity and a lack of sexual self-control; this was especially prominent amongst HIV-positive FGD participants. This may highlight a different health counselling need in women living with HIV with regards to cervical screening.

Women were clear that condoms offered protection (82.7, 94.6%), and HIV-positive participants seemed to have adopted the HIV prevention message “use a condom every time you have sex” as applicable to HPV.

A third of women found screening embarrassing, as is commonly reported [11, 31, 43]. Self-sampling is suggested as a potential means of managing this barrier [31]. Less than 10% of women found screening painful; an aspect that could be shared with unscreened women, as fear of pain can be a reason for screening-avoidance [43–45]. Women expressed other causes of fear and anxiety such as “scary” public health campaigns, finding out about HPV and cervical cancer for the first time, and waiting for their results. This is consistent with research in high-income countries where HPV-DNA testing and cervical cancer screening have been shown to generate anxiety and distress [46–48]. Having one’s information needs met was the same protective factor against screening-anxiety mentioned by these women as women in high-income-settings [24, 40, 49]. A UK study found that women with poorer access to information and of lower educational status reported more anxiety about HPV-DNA testing results, which echoes our finding that women with less education were more likely to worry about test results [24].

Over 90% of women in both groups understood that cervical cancer is preventable (90.8% vrs. 94.6%) among HIV negative compared with HIV positive women, and that screening should be done even if asymptomatic (94.7% vrs. 94.6%). Women also expressed that screening allows early detection and treatment of problems. This contrasts with a qualitative study in Ghana, where women strongly expressed that screening was only necessary if one had symptoms, again showing the effectiveness of study counselling [50].

Cost is a reported barrier to screening in other studies in SSA [15, 43], and this was also evident in our study. Some women had not had previous screening due to cost, and a number indicated that they would have screening again only if deemed medically “necessary” and at a lower price.

### Strengths and limitations

This study is the first in West Africa that examines the knowledge and experiences of women who have undergone cervical screening with two screening methods (HPV-DNA testing and cervical cytology). It provides a unique perspective on the impact of health education from screening, and psychological experiences of screening in women of mixed HIV-status. Both the questionnaire and scoring system used were developed specifically for this study and were unvalidated. There was no pre-existing validated tool suitable for use with this particular population.

Furthermore, we did not assess knowledge levels before participation in the study and cannot assume that knowledge “gains” were made, although it seems likely based on participant statements during the FGDs and comparison with existing research in similar settings. In addition, since these women had undergone screening and received some education from healthcare workers, it is acknowledged that they do not necessarily reflect the general population hence their knowledge may not represent women across Ghana and differences between HIV-positive and negative women needs to be explored further.

Ideally, questionnaire responses would have been used to develop the FGD topic-guide, and more FGDs should have been conducted to verify if saturation was achieved. However, this was not possible due to time constraints. As a result, there were aspects of each study component that were not explored in the other, causing some loss of cohesiveness.

## Conclusions

This study showed that whilst knowledge of HPV and cervical cancer in women who had participated in a screening study was good, specific misconceptions still existed. A standardised education tool explaining cervical screening and specifically HPV-DNA testing in Ghana may be needed, which should be made accessible to women with low formal education, and may take into account the different needs of HIV-positive women. Public health messaging should take into account the issues of fear and economic barriers to accessing services. Further research into the psychological effects of cervical cancer screening on women in Ghana should be undertaken, in order to strengthen the knowledge base to improve screening, especially as HPV-DNA testing becomes more widely used.

## Data Availability

The datasets generated and/or analysed during the current study is available from the corresponding author on reasonable request.

## Abbreviations

†: Denotes use of Fisher’s exact test
‡: Denotes use of Chi-squared test
95% CI: 95% Confidence Interval
CCTH: Cape Coast Teaching Hospital
CIN: Cervical intraepithelial neoplasia
FGD(s): Focus group discussion(s)
HIV: Human immunodeficiency virus
HPV: Human papillomavirus
OR(s): Odds ratio(s)
SD: Standard deviation
SSA: Sub-Saharan Africa
